# Assessing the Radiological and Functional Outcomes of Colles' Cast Versus Functional Position Cast Immobilization in the Conservative Treatment of Distal End of Radius Fractures

**DOI:** 10.7759/cureus.63492

**Published:** 2024-06-30

**Authors:** Vijay Ranjan, Udit Agrawal, Gautam Chatterji, Sourav Shukla, Vaibhav B K., Digvijay Mishra

**Affiliations:** 1 Orthopedics and Trauma, The Bone and Joint Trauma Center, Madhubani, IND; 2 Pediatric Orthopedics, King George's Medical University, Lucknow, IND; 3 Orthopedics, People’s College of Medical Sciences and Research Centre, Bhopal, IND; 4 Orthopedic Surgery, Vivekananda Polyclinic and Institute of Medical Sciences, Lucknow, IND; 5 Orthopedic Surgery, Ramakrishna Sewashram Hospital, Mirzapur, IND

**Keywords:** functional position cast, colle's cast, distal end radius fracture, functional, radiological

## Abstract

Introduction: The distal end radius fracture represents a prevalent orthopedic condition that affects individuals across various age groups, commonly resulting from falls onto outstretched hands. Ongoing research endeavors have delved into diverse methodologies for addressing this condition, encompassing conservative and operative modalities, yielding variable outcomes. While the literature extensively delineates numerous mobilization approaches, such as the functional position cast and Colle's cast, scant comparative studies evaluate these immobilization forms. Consequently, our study sought to holistically appraise and compare the radiological and functional outcomes associated with Colles' cast and functional position cast immobilization in managing distal end radius fractures.

Method: This retrospective study was conducted at a tertiary trauma center over two years, from October 2018 to September 2020. Data was collected from medical records with prior approval from the institutional ethics committee. The study included 64 patients, all above the age of 40, who suffered from distal end radius fractures and received conservative management. Patients with bilateral distal end radius fractures, associated ipsilateral limb injury, open or comminuted fractures, or inadequate medical records were excluded. The patients were divided into two groups based on the type of cast immobilization: group I comprised 30 patients managed with Colles' cast immobilization (volar-flexion and ulnar deviation position). In contrast, group II consisted of 34 patients with functional position cast immobilization (immobilization with dorsiflexion). The clinical (including pain, thumb swelling, finger swelling, finger stiffness, shoulder stiffness, and tenderness), radiological (including radial deviation, radial height, and volar tilt), and functional (range of motion (ROM), and disability of arm, shoulder, and hand (DASH)) outcomes were evaluated at 6, 12, and 24 weeks. The data analysis was conducted using the IBM SPSS Statistics for Windows, Version 25 (Released 2017; IBM Corp., Armonk, New York, United States). The chi-square test, independent samples t-test, and paired t-test were employed to analyze and compare radiological and functional outcomes between the two groups. A p-value of less than 0.05 indicated a statistically significant association.

Results: The radiological parameters, including volar tilt, radial inclination, and radial shortening, were derived from the medical records at various points: pre-reduction, post-reduction, 6-week follow-up, 12-week follow-up, and 24-week follow-up. Upon assessing these parameters, no statistically significant variance was observed between the two groups at specified time points. Comparison of the ROM between the two groups unveiled noteworthy results, indicating superior outcomes in the functional cast group at 6 and 12 weeks as opposed to the Colles' cast group. Grip strength assessment at the 24-week follow-up demonstrated statistically significant differences, with the functional cast group displaying enhanced grip strength.

Conclusion: Our study revealed comparable radiological parameters between the two cohorts, yet it demonstrated a notable enhancement in both the ROM and functional outcomes in those subjected to functional cast positioning. These findings underscore the potential advantages of functional immobilization in bolstering patient rehabilitation.

## Introduction

Distal radius fractures are a prevalent orthopedic condition, with an incidence rate of approximately 1 in 10000, constituting about 17% of all fractures [1,2]. Over the past four decades, the incidence of these fractures has exhibited an upward trajectory, a trend attributed to various factors [3,4]. Key factors include evolving dietary patterns impacting bone metabolism, epigenetic influences on osteoporotic diseases, and heightened participation in sports-related activities [5-7]. These fractures are particularly prevalent among the elderly due to their propensity for osteoporosis [5,8]. Research indicates a higher incidence in women over 50 than men in the same age group [8,9]. Notably, treatment of distal radius fractures is often accompanied by various complications such as reflex sympathetic dystrophy (RSD), malunion, restricted joint mobility, and persistent pain [10,11]. Elderly patients, in particular, exhibit a heightened likelihood of mortality within seven years following their initial distal radius fracture [12].

A diverse array of treatment modalities is utilized to address distal end radius fractures, with the primary objective of reinstating articular congruity, radial length, and overall upper extremity function [13]. Medical literature has detailed various conservative treatment options, encompassing functional bracing, wrist immobilization in dorsiflexion, neutral or palmar flexion, and pronation or supination [14-17]. The original reduction, initially elucidated by Cotton as volar flexion and ulnar deviation [18], was subsequently popularized by Charnley [19], building upon Cotton's foundational work in 1910. The application of this particular position is posited to preserve the reduction utilizing the ligamentotaxis principle [20].

In clinical practice, the positioning of the wrist in flexion-ulnar deviation (Colles') may lead to the tightening of the joint extensor tendons, resulting in inappropriate finger flexion during treatment. Immobilization of the wrist in palmar flexion can detrimentally affect hand function, as dorsiflexion at the wrist is essential for adequately rehabilitating fingers [17]. This position presents challenges concerning degenerative joints, particularly in the elderly demographic, which is susceptible to stiffness. The optimal hand function position involves wrist dorsiflexion, thereby giving rise to the widely employed immobilization method known as the functional cast position. This approach immobilizes the wrist in 0-20 degrees of dorsal angulation, purportedly maintaining improved functional capability while facilitating wrist and hand rehabilitation.

This study aims to assess the radiological and functional outcomes of Colles' cast compared to functional position cast immobilization techniques in the conservative treatment of distal end radius fractures.

## Materials and methods

This retrospective study was conducted at a tertiary trauma center over two years, from October 2018 to September 2020. Data was collected from medical records with prior approval from the Vivekananda Polyclinic and Institute of Medical Sciences Institutional Ethics Committee (VPIMS/ME/NBE/Th.Proto/2018). The study included 64 patients, all above the age of 40, who suffered from distal end radius fractures and received conservative management in the form of cast immobilization. Patients with bilateral distal end radius fractures, associated ipsilateral limb injury, open or comminuted fractures, or inadequate medical records (missing follow-up visits, radiographs, and measurements) were excluded. The patients were divided into two groups based on the type of cast immobilization. Group I comprised 30 patients managed with Colles' cast immobilization (volar-flexion and ulnar deviation position). In contrast, group II consisted of 34 patients with functional position cast immobilization (wrist immobilization in dorsiflexion).

**Figure 1:**
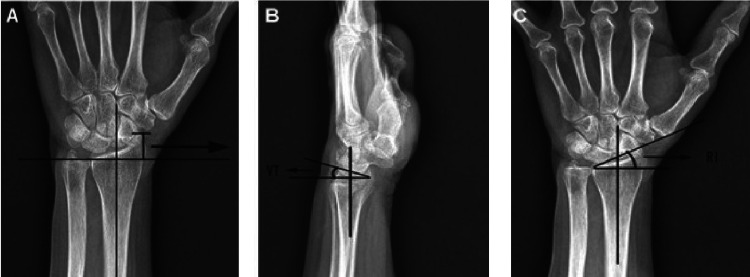
Methods of measuring radiographic parameters. A) radial height; B) volar tilt; C) radial inclination

Our institution consistently conducts follow-up examinations on these patients at 6, 12, and 24 weeks post-intervention. Consequently, our data collection is limited to these specific time points. Radiological evaluations were routinely performed pre- and post-reduction at 6, 12, and 24 weeks. The observed radiological parameters encompass radial deviation, radial height, and volar tilt, all measured using standard anteroposterior and lateral views and subsequently compared with the unaffected side. At the 24-week follow-up, radiological outcomes were assessed utilizing Sarmiento's modification of Lindstrom criteria [21]. A reference line was delineated along the central longitudinal radius axis in anteroposterior and lateral radiographs to determine radial height. A perpendicular line was drawn to the central axis, and another line crossed the ulnar and radial margins of the radial articular surface in the anteroposterior view. The perpendicular distance between the initial two lines was quantified in millimeters (mm) and documented as the radial height (Figure 1A). The angle formed by the third line and the first perpendicular line denoted radial deviation in degrees (Figure 1C). Volar/dorsal tilt was computed by tracing a perpendicular line to the central axis, in addition to a line passing through the ulnar and radial margins of the radial articular surface in the lateral view. A positive angle indicated a volar tilt, while a negative angle signified a dorsal tilt (Figure 1B).

The clinical assessments were conducted 6, 12, and 24 weeks post-onset. The parameters included pain, thumb swelling, finger swelling, finger stiffness, shoulder stiffness, and tenderness. Grip strength, range of motion (ROM), and the disability of arm, shoulder, and hand (DASH) [22] were utilized as determinants of wrist functional outcome post-distal radius fracture. ROM encompassed wrist flexion/extension measurements, supination, pronation, ulnar deviation, radial deviation, and circumduction. Functional outcomes were evaluated at 6, 12, and 24 weeks. The Demerit score [23] assessed functional outcomes during the 24-week follow-up. Additionally, grip strength was measured in both groups at the 24-week follow-up.

The data analysis was conducted using the IBM SPSS Statistics for Windows, Version 25 (Released 2017; IBM Corp., Armonk, New York, United States). The chi-square, independent samples t-test, and paired t-test were employed to analyze and compare radiological and functional outcomes between the two groups. A p-value of less than 0.05 indicated a statistically significant association.

## Results

During the period from October 2017 to September 2018, a total of 64 participants were included in the research study. They were categorized into two cohorts based on the type of cast administered: group I, comprising patients treated with Cole's cast, and group II, consisting of patients treated with a functional cast. Group I comprised 30 participants, while group II comprised 34. Data analysis utilized the information extracted from the participant's medical records. Table 1 presents the baseline characteristics of the enrolled participants as documented in the medical records.

**Table 1:** Baseline characteristics of two groups

Characteristics	Group I	Group II
Number of patients	30	34
Age (years), mean (range)	58.4 (41-88)	59.2 (42-86)
Sex (female/male)	17/13 (56.66%/43.33%)	22/12 (64.70%/35.29%)
Dominant hand (right/left)	27/3 (90%/10%)	30/4 (88.23%/11.76%)
Fracture side (right/left)	22/8 (73.33%/26.66%)	24/10 (70.58%/29.41%)
Fracture in the dominant hand	25 (83.33%)	28 (82.35%)
Extra-/intra-articular fractures	16/14 (53.33%/46.66%)	18/16 (52.94%/47.05%)

Radiological evaluation

The radiological parameters, including volar tilt, radial inclination, and radial shortening, were derived from the medical records at various points: pre-reduction, post-reduction, 6-week follow-up, 12-week follow-up, and 24-week follow-up. Upon assessing these parameters, no statistically significant variance was observed between the two groups at specified time points. Notably, when Sarmiento's modification of Lindstrom criteria was employed at the 24-week follow-up, 13.33% of patients in group I demonstrated excellent results compared to 8.82% in group II. It should be noted that this variance was not found to be statistically significant (Table 2).

**Table 2:** Radiological parameters among the two groups at respective follow-ups

Radiological parameters		Group I		Group II		Statistical analysis	
Volar tilt (degrees)		Mean	SD	Mean	SD	T-test	p-value
	Pre-reduction	-20.31	3.89	-18.96	5.19	1.177	0.244
	Post-reduction	6.84	3.23	6.78	3.99	0.066	0.948
	6 weeks follow-up	10.05	1.07	10.68	2.48	1.319	0.192
	12 weeks follow-up	10.21	2.1	10.71	4.35	0.586	0.56
	24 weeks follow-up	10.2	3.24	10.73	4.38	0.55	0.584
Radial inclination (degrees)	Pre-reduction	13.11	3.95	12.84	4.58	0.253	0.801
	Post-reduction	14.81	4.99	15.12	3.21	0.296	0.768
	6 weeks follow-up	20.06	4.27	22.06	4.31	1.865	0.067
	12 weeks follow-up	20.62	5.82	22.34	5.76	1.188	0.239
	24 weeks follow-up	20.76	4.73	22.38	5.36	1.282	0.205
Radial Shortening (mm)	Pre-reduction	5.48	2.34	6.37	3.18	1.275	0.207
	Post-reduction	7.62	3.68	8.28	3.32	0.753	0.454
	6 weeks follow-up	11.3	1.38	11.28	1.16	0.063	0.950
	12 weeks follow-up	11.64	1.34	11.3	4.07	0.449	0.655
	24 weeks follow-up	11.7	3.88	11.62	3.94	0.082	0.935
Sarmiento's modification of Lindstrom criteria at 24 weeks follow-up		N	%	N	%	χ^2^	p
	Excellent	3	10%	4	11.76%	0.582	0.901
	Good	16	53.33%	20	58.82%		
	Fair	9	30%	10	29.41%		
	Poor	2	6.66%	1	2.94%		

Clinical evaluation

During each routine follow-up, patients underwent evaluation for clinical parameters, including pain, swelling of the thumb and fingers, stiffness of fingers and shoulder, and tenderness. Patients with Colles' cast exhibited a higher incidence of pain (66.66%) and tenderness (63.33%) at the six-week follow-up. However, statistical analysis revealed that the differences between the two groups were statistically insignificant at the 6-week, 12-week, and 24-week follow-up assessments (see Table 3).

**Table 3:** Clinical assessment among two groups at respective follow-ups

		Group I		Group II		Statistical analysis	
1^st ^follow-up (6 weeks)		N	%	N	%	χ^2^	p-value
	Pain	20	66.66%	19	55.88%	1.626	0.798
	Swelling (thumb)	11	36.66%	5	14.70%	0.011	0.083
	Swelling (fingers)	14	46.66%	7	20.58%	0.006	0.062
	Stiffness of fingers	4	13.33%	1	2.9%	0.042	0.162
	Stiffness of shoulder	3	10%	4	11.76%	1.026	0.689
	Tenderness	19	63.33%	14	41.17%	0.072	0.211
2^nd^ follow-up (12 weeks)	Pain	12	40%	7	20.58%	0.047	0.171
	Swelling (thumb)	4	13.33%	1	2.9%	0.042	0.162
	Swelling (fingers)	5	16.66%	1	2.9%	0.012	0.086
	Stiffness of fingers	2	6.66%	1	2.9%	0.581	0.554
	Stiffness of shoulder	2	6.66%	2	5.88%	0.000	1.000
	Tenderness	13	43.33%	9	26.47%	0.141	0.292
3^rd^ follow-up (24 weeks)	Pain	3	10%	1	2.94%	0.15	0.302
	Swelling (thumb)	2	6.66%	0	0%	0.036	0.151
	Swelling (fingers)	0	0%	0	0%	0.000	1.000
	Stiffness of fingers	1	3.33%	0	0%	0.163	0.313
	Stiffness of shoulder	1	3.33%	1	2.94%	0.000	1.000
	Tenderness	7	23.33%	3	8.82%	0.045	0.168

Functional outcome

Before fracture reduction in group I, 15 patients (50%) reported severe wrist pain and difficulty, 10 patients (33.3%) experienced moderate pain, and 5 patients (16.66%) reported mild pain. In group II, 18 patients (52.9%) reported severe pain, 11 patients (32.35%) had moderate pain, and 5 patients (14.7%) had mild pain.

Following six weeks, both groups displayed reduced pain and improved functionality, with patients in group II exhibiting significant improvement. During this period, 14 patients in group II scored between 30 and 40 points on the DASH scale, 18 scored between 20 and 29 points, and 2 scored 50 points or higher. In group I, 13 patients scored 30 to 40 points, 20 scored 20 to 29, and 1 scored 50 or higher.

After 12 weeks, three patients (10%) treated with a Colle's cast and six (17.6%) treated with a functional position cast regained functional hand use without difficulty. Fifteen patients in group I and 21 in group II reported mild to moderate difficulty using the affected hand. Furthermore, 12 patients in the Colles' cast group and 7 patients in the functional position cast group experienced severe pain after 12 weeks.

At 24 weeks, 16 patients (53.33%) treated with a Colle's cast and 26 (76.47%) treated with a functional position cast regained functional hand use without difficulty. In addition, 11 patients in group I and 7 in group II reported mild to moderate difficulty using the affected hand. Notably, three patients in the Colles' cast group and one patient in the functional position cast group experienced severe pain after 24 weeks.

The mean values of DASH among both groups exhibited no significant difference (p = 0.124) at the 1st follow-up. Still, statistically significant differences were observed at the 2nd (12 weeks) (p = 0.001) and 3rd (24 weeks) (p = 0.026) follow-ups (Table 4).

**Table 4:** The comparison of demerit point scores among two groups at 24 weeks of follow-up

Demerit point score system at 24 weeks follow-up	Group I (N = 30)	Group II (N = 34)	Statistical analysis
Excellent (0-2)	3 (10%)	4 (11.76%)	p = 0.484
Good (3-8)	13 (43.33%)	19 (55.88%)	
Fair (9-20)	10 (33.33%)	8 (23.52%)	
Poor (>20)	4 (13.33%)	3 (8.82%)	

The Demerit point score system was computed at the 24-week follow-up for both groups, with more patients in group I demonstrating excellent results. However, this outcome was not deemed statistically significant (p-value = 0.484) (Table 5).

**Table 5:** Mean values of DASH score among two groups at respective follow-up

	DASH score (mean ± SD)		
	1^st^ follow-up (6 weeks)	2^nd ^follow-up (12 weeks)	3^rd^ follow-up (24 weeks)
Group I	30.38 ± 6.35	22.13 ± 18.75	16.88 ± 14.22
Group II	30.91 ± 9.34	20.59 ± 8.28	13.34 ± 9.22
p-value	0.124	p < 0.001	0.026

ROM

Dorsiflexion

A statistical comparison revealed a statistically significant difference (p ≤ 0.05) between the two groups at all follow-up assessments.

Palmar Flexion

A statistically significant difference in palmar flexion between the two groups was observed at the 1st and 2nd follow-up (p ≤ 0.001) but not at the 3rd follow-up (p ≤ 0.639).

Ulnar Deviation

On applying the student's t-test, we indicated a statistically significant difference between the two groups in deviation at the 1st (p ≤ 0.001) and 2nd (p ≤ 0.001) follow-ups but not at the 3rd follow-up (p ≤ 0.507).

Radial Deviation

Statistically significant differences between the two groups in deviation were observed at the 1st follow-up (p ≤ 0.001) but not at the 2nd (p ≤ 0.120) and 3rd follow-up (p ≤ 0.773).

Supination

The analysis demonstrated statistically significant differences between the two groups in terms of supination at the 1st (p ≤ 0.001) and 2nd follow-up (p ≤ 0.001) but not at the 3rd follow-up (p = 0.233).

Pronation

Similarly, statistically significant differences between the two groups in terms of pronation were noted at the 1st (p ≤ 0.001) and 2nd follow-up (p = 0.001) but not at the 3rd follow-up (p = 0.216).

Grip Strength

Grip strength was assessed between both groups at 24 weeks. The mean grip strength in Colles' cast was 56 ± 12.24, while the functional position cast provided a mean grip strength of 62.69 ± 12.13. Employing a t-test for significance revealed a notable difference between the two groups (p ≤ 0.033). The functional position cast exhibited superior grip strength compared to the Colles' cast (Table 6).

**Table 6:** Range of motion and grip strength comparison among two groups at respective follow-up

Range of motion		Group I		Group II		Statistical analysis	
Dorsiflexion (degrees)		Mean	SD	Mean	SD	T-test	p-value
	1^st^ follow-up	44.78	6.38	54.06	3.41	7.264	<0.001
	2^nd^ follow-up	58.78	3.93	63.69	6.65	3.592	0.001
	3^rd^ follow-up	68.66	9.95	62.34	8.73	2.431	0.018
Palmar flexion (degrees)	1^st^ follow-up	49.97	3.290	65.38	2.978	19.639	<0.001
	2^nd^ follow-up	65.09	4.748	71.97	8.630	3.948	<0.001
	3^rd^ follow-up	71.28	15.778	69.34	17.034	-0.472	0.639
Ulnar deviation (degrees)	1^st^ follow-up	15.28	2.20	22.56	1.90	14.159	<0.001
	2^nd^ follow-up	23.50	1.33	26.28	3.55	4.155	<0.001
	3^rd^ follow-up	24.34	4.97	25.19	5.13	0.668	0.507
Radial deviation (degrees)	1^st^ follow-up	6.91	1.60	13.41	2.07	14.046	<0.001
	2^nd^ follow-up	12.53	1.79	16.41	2.58	1.576	0.001
	3^rd^ follow-up	16.88	2.15	16.69	2.96	-0.290	0.773
Supination (degrees)	1^st^ follow-up	49.75	3.22	65.56	3.17	19.781	<0.001
	2^nd^ follow-up	64.50	3.20	70.91	7.91	4.249	<0.001
	3^rd^ follow-up	66.28	9.12	69.25	10.56	1.204	0.233
Pronation (degrees)	1^st^ follow-up	49.63	3.37	64.94	3.74	17.209	<0.001
	2^nd^ follow-up	65.59	4.14	71.09	7.87	3.501	0.001
	3^rd^ follow-up	65.03	9.93	68.16	10.06	1.250	0.216
		Mean	SD	Mean	SD	t	P
Grip strength at final (24 weeks) follow-up		56.03	12.24	62.69	12.13	-2.185	0.033

## Discussion

Colles' fracture ranks as one of the most prevalent fractures among the elderly demographic, with a higher incidence noted among female patients, as corroborated by the findings of Gnawali [24]. This predilection may be attributed to the established association between Colles' fracture and osteoporosis in women, who exhibit a heightened susceptibility to sustaining such injuries from falls while engaged in domestic activities as opposed to road traffic incidents [25]. Managing Colles' fractures presents challenges, with no definitive consensus on optimal treatment modalities. Conservative management emerges as a prudent option, affording favorable clinical outcomes. Sarmiento recommends immobilization in supination to mitigate the deforming force exerted by the brachioradialis, thus minimizing the risk of reduction loss [14]. At the same time, Wahlström advocates immobilization in pronation, citing the pronator quadratus as a deforming force contributing to reduction loss [15].

In our study, upon assessing the radiological parameters, no significant differences were observed between the two cohorts at any point in time about volar tilt, radial inclination, and radial shortening. This finding is consistent with the existing literature, as evidenced by similar results reported in the studies conducted by Gupta [26] and Grafstein et al. [27]. Radiological outcomes were appraised using Sarmiento's modification of Lindstrom criteria at the 24-week follow-up. No statistically significant differences were noticed, thus reflecting alignment with the findings of Gupta et al. [26].

Regarding the clinical evaluation, it was noted that patients with Colles' cast exhibited a higher incidence of pain (66.66%) and tenderness (63.33%) during the six-week follow-up period. Nonetheless, statistical analysis delineated the insignificance of intergroup disparities at the six-week and all subsequent follow-up intervals, consistent with the findings of Grle et al. [28] and Grafstein et al. [27].

The functional outcomes were evaluated using the DASH score, showing no significant difference (p = 0.124) at the initial follow-up. However, notable variances were evident at the subsequent follow-up assessments at 12 weeks (p = 0.001) and 24 weeks (p = 0.026), aligning with the research of Blatter et al. [29] and Rajan et al. [30]. The Demerit point score system was computed at the 24-week follow-up for both groups. While more patients in group 2 exhibited excellent results, this disparity was not deemed statistically significant (p = 0.484), in line with Gupta et al.'s findings [26].

Comparison of the ROM between the two groups unveiled noteworthy results, indicating superior outcomes in the functional cast group at 6 and 12 weeks as opposed to the Colles' cast group. However, by the 24-week mark, none of the metrics revealed statistically significant distinctions except for dorsiflexion. These outcomes corroborate prior studies demonstrating improved results in the functional cast group [28-30]. Grip strength assessment at the 24-week follow-up showed statistically significant differences, with the functional cast group displaying enhanced grip strength, consistent with the prior study by Rajan et al. [30].

Several limitations are associated with our study, encompassing its retrospective design, a relatively small sample size, and a brief follow-up period.

## Conclusions

The selection of the appropriate form of immobilization following the reduction of the distal end of the radius continues to be a matter of ongoing debate. Our study revealed comparable radiological parameters between the two cohorts. Yet, it demonstrated a notable enhancement in both the ROM and functional outcomes in those subjected to functional cast positioning. These findings underscore the potential advantages of functional immobilization in bolstering patient rehabilitation. Such studies are necessary as they provide valuable insights for clinical practice and facilitate the adoption of more effective conservative management strategies for distal radius fractures. Nevertheless, further research involving larger cohorts and extended follow-up periods is imperative to authenticate these findings.
